# Effect of a Traditional Chinese Medicine Formula (CoTOL) on Serum Uric Acid and Intestinal Flora in Obese Hyperuricemic Mice Inoculated with Intestinal Bacteria

**DOI:** 10.1155/2020/8831937

**Published:** 2020-12-23

**Authors:** Yan Gao, Jing Sun, Yi Zhang, Tiejuan Shao, Haichang Li, Meijiao Wang, Li Zhang, Hua Bian, Chengping Wen, Zhijun Xie, Huiqing Lv

**Affiliations:** ^1^Zhejiang Chinese Medical University, Hangzhou, Zhejiang, China; ^2^The Second Clinical Medical College of Zhejiang Chinese Medical University, Hangzhou, Zhejiang, China; ^3^Hangzhou Traditional Chinese Medical Hospital, Hangzhou, Zhejiang, China; ^4^Henan Key Laboratory of Zhang Zhongjing Formulae and Herbs for Immunoregulation, Nanyang Institute of Technology, Nanyang, Henan, China

## Abstract

CoTOL is a traditional Chinese medicine (TCM) formula in clinics for treating gout and hyperuricemia, especially in obese patients with recurrent attacks. However, fewer studies have investigated how CoTOL impacts the intestinal flora in reducing uric acid. In the present, we analyze the bacteria targeted by ingredients of CoTOL and evaluate the effects of CoTOL on uric acid and intestinal flora in a mice model of obese hyperuricemia inoculated with xanthine dehydrogenase- (XOD-) producing bacteria, *Streptococcus faecalis*. Firstly, ingredients of herbs in CoTOL and gene target by these ingredients were retrieved from TCMID 2.0, and these genes were screened by DAVID Bioinformatics Resources 6.8, deciphered to retrieve the bacteria. Then, 3-4-week-old male C57bl/6j mice were randomly divided into 6 groups and fed with high fat diet for 8 weeks up to obesity standard. The mice were inoculated intragastrically with 5 × 10^9^ CFU *Streptococcus faecalis* 3 times at the 5^th^, 6^th^, and 7^th^ week and intragastrically administrated with uricase inhibitor, potassium-oxonate (PO, 250 mg/kg), to induce hyperuricemia at the 8^th^ week, once a day for 7 consecutive days, respectively (IB model). IB model plus CoTOL (0.4 ml/20g) and allopurinol (40 mg/kg) were administrated by gavage at the 5^th^ week, once a day for 4 weeks. The feces and blood in each group were sampled at the 4^th^ and 8^th^ week. With no bacteria inoculation, CoTOL, allopurinol, and blank group were treated with CoTOL and allopurinol or water, respectively. 44 species of bacteria (i.e., *Enterococcus faecalis, Streptococcus,* etc.) genes were targeted by 6 ingredients of 6 herbs in CoTOL. Inoculation with *Streptococcus faecalis* significantly caused the elevation of uric acid and the change of intestinal flora structure, whereas treatment with CoTOL signiﬁcantly increased the abundance of *Akkermansia* and those of *Bacteroides* and *Alloprevotella* decreased. Furthermore, CoTOL exhibited a unique effect on reducing weight unobserved in allopurinol intervention. The present study, for the first time, demonstrated that CoTOL has beneficial effects on hyperuricemia and overweight, which may be attributed to regulating material metabolism and improving the structure or function of intestinal flora. Thus, CoTOL may be a promising therapy for hyperuricemia and overweight in chronic gout management and can be integrated with conventional treatments.

## 1. Introduction

Gout is a common chronic purine metabolic disorder [1] characterized by hyperuricemia and recurrent gouty arthritis, which cause disﬁgurement, bone destruction, and disability. Gout affects almost 3.9% of adults in the USA, 2.5% in the UK, and 2.2% in China [2]. The prevalence of gout is rising globally, especially among individuals with chronic diseases such as hypertension, chronic kidney disease, diabetes, obesity, congestive heart failure, and myocardial infarction [3]. Obesity, hypertension, and diuretic use are the main risk factors for incident gout [4, 5].

Xanthine oxidase (XOD), a key oxidative enzyme, could oxidize hypoxanthine and xanthine to uric acid in the purine catabolic pathway [6]. XOD-producing bacteria are extensively distributed in mammal guts, especially *Clostridium acidurici*, *Clostridium purinilyticum*, and *Enterococcus faecalis (*i.e., *Streptococcus faecalis)* [7], which exhibit some physiological roles [8, 9]. Studies have shown that a third of uric acid is excreted from the gut or decomposed by microbes in the gut and the decrease of uric acid function in intestinal microorganism is an important cause of hyperuricemia and gout [10, 11]. Potassium-oxonate (PO) is usually used as a competitive uricase inhibitor to develop hyperuricemia in rats by inhibiting uricase and to evaluate possible therapeutic agents.

Chinese herbal medicine has unique advantages in the treatment of gout [12]. Our previous multicenter double-blind randomized controlled clinical trial has demonstrated that Traditional Chinese medicine (TCM) formula, CoTOL, has hypouricemic and arthritis relapse-reducing effects on intercritical and chronic gout with less adverse reactions. CoTOL is a clinical experienced formula, which has been widely used to treat obese patients associated with complicated pathologic states of gout in China. Furthermore, CoTOL contains many complex ingredients which target associated genes/proteins or intestinal flora.

In the present study, we used the network pharmacological method as described in a previous study [13] to analyze a large number of human or bacterial genes/proteins associated with complex ingredients of this TCM formula and to predict its effects on gout associated proteins or intestinal flora. Furthermore, we constructed an obese hyperuricemic mice model and further inoculated intragastrically with *Streptococcus faecalis* (a type of XDH-producing bacteria). Our purpose is to evaluate the efficacy of CoTOL in reducing weight and uric acid by regulating intestinal flora. We also clarified its correlation of uric acid and intestinal flora based on the mice model.

## 2. Methods

### 2.1. Preparations of CoTOL and Reagents

CoTOL is composed of several medicinal herbs (Table 1) which were identified by Dr. Min Qian and obtained from Medical Pieces Co., Ltd., of Zhejiang Chinese Medical University (Hangzhou, Zhejiang, China). All herbs were grounded and extracted twice with deionized water under reflux for 2 h. The filtered extracts were concentrated to a decoction (1.82 g/ml) using a rotary evaporator. As reported in our previous papers [14, 15], we have identified the main ingredients of CoTOL which are diosgenin, quercetin, and luteolin by rapid liquid chromatography (RPLC) for quality control. Allopurinol tablets were obtained from Tianjin Pharmaceutical Co., Ltd. (Chinese Medicine Standard Number H20033683). Potassium-oxonate (PO) was purchased from Sigma Chemicals (St. Louis, MO, USA). Fecal DNA extraction kits were obtained from Shanghai Jierui Bioengineering Co., Ltd., *Streptococcus faecalis* (our laboratory preservation). The deionized water was purified by a Milli-Q system (Millipore, Bedford, USA). All other reagents used were of analytical grade. The protocol was approved by our departmental ethics committee.

### 2.2. Bacterial Genes Targeted by Ingredients of CoTOL

As described in our previous study [13], genes targeted by ingredients of herbs in CoTOL were retrieved from TCMID 2.0 [16]. We further retrieved the bacteria containing these genes with a comprehensive set of functional annotation tools of DAVID Bioinformatics Resources 6.8 [17]. Based on Cytoscape (bioinformatics software) V3.3.0 [18], we tried to analyze the data and construct the visualized networks of herbs, ingredients of herbs, bacterial genes, and bacteria.

### 2.3. Animals

3-4-week old male C57bl/6j mice were obtained from the Experimental Animal Center of Zhejiang Chinese Medical University (SCXK (Yu)-2005–3001, Zhejiang Province, China). The animals were housed in 8-hour illumination per day, room temperature 22°C, relative humidity 55 ± 10% fed with plentiful food and water. They were treated in accordance with the Regulations of Animal Administration issued by the State Committee of Science and Technology of People's Republic of China.

### 2.4. Experimental

#### 2.4.1. Obese Hyperuricemic Mice Inoculated with Intestinal Bacteria

**Figure 1:**
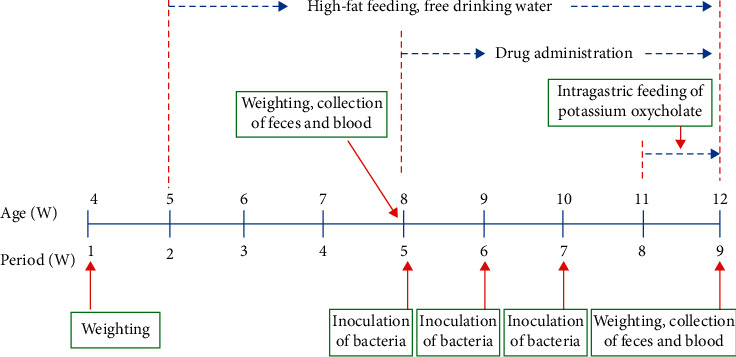
Animal model making process.

The mice were randomly divided into six groups as follows: IB model group, IB model plus CoTOL group, IB model plus allopurinol group, CoTOL group, allopurinol group, and blank group. After one week of adaptive feeding, the mice were fed with high fat forage (fat content about 36%) of C57bl/6j for 8 weeks. Weight gain of more than 20% at the end of the 8^th^ week is up to the obesity model standard. The mice in IB model groups were inoculated intragastrically with 5 × 10^9^ CFU XOD-producing bacteria (*Streptococcus faecalis*) 3 times at the 5^th^, 6^th^, and 7^th^ week, respectively. The mice in IB model plus CoTOL group and IB model plus allopurinol group were intragastrically administrated with 0.4 ml/20g of CoTOL or 40 mg/kg allopurinol solution, respectively, once a day from the beginning of the 5^th^ week. Without being inoculated intragastrically with bacteria, the mice in CoTOL group, allopurinol group, and blank group were intragastrically administrated with CoTOL. We determined the main ingredients of CoTOL to control its quality using rapid liquid chromatography. Meanwhile, except for mice in blank group, the mice in the other 5 groups were intragastrically administrated with 250 mg/kg PO from the beginning of the 8th week, once a day for 7 consecutive days. At the 4^th^ and 8^th^ weekend, the feces and blood of mice in each group were collected and stored at −80°C for the following detection (Figure 1).

#### 2.4.2. Measurement of Body Weight and Serum Uric Acid

After adaptive feeding for one week, the C57bl/6j mice were fed with high fat forage (fat content about 36%) for 8 weeks up to the obesity model standard. The mice weight was gained at the 4^th^ and 8^th^ week, respectively. The bloods in each group were collected at the 4^th^ and 8^th^ weekend to measure the serum uric acid levels by Olympus AU 640 automatic biochemical analyzer.

#### 2.4.3. Intestinal Flora Analysis

Experimental process of DNA extraction and identification of feces were performed with the methods as described in a previous study [19].

## 3. Statistics

The experimental data were expressed by mean standard deviation. The test standard was *p* < 0.05 by spss18.0 for windows software, the variance analysis was used when the variance was homogeneous among multiple groups, the LSD-t method was used for pairwise comparison between groups, and the Kruskal–Wallis H test was used when the variance between multiple groups was uneven. The relationship between the number change of operational taxonomic units (OTU) and the similarity value of clustering was drawn by research software, the data were analyzed and counted by QIIME, Mothur, and other software when the similarity was 97%, the graph was made by *R* software, and the statistical results of species taxonomy were plotted.

The OTU cluster analysis, alpha diversity analysis, species classification, differential analysis of flora, and function prediction and analysis were performed with the methods as described in a previous study [19].

## 4. Results

### 4.1. Bacterial Genes Targeted by Ingredients of CoTOL

**Figure 2:**
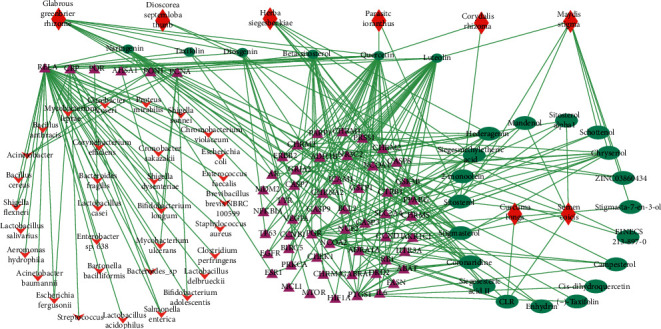
Bacterial targeted by ingredients of CoTOL. A total of 25 ingredients of the 8 herbs of CoTOL are targeted, of which 6 ingredients of 6 kinds of herbs target 44 bacteria. The red prismatic nodes represent herbs of CoTOL, the green oval nodes represent herbal ingredients, the pink triangular nodes represent the corresponding targets of the herbal ingredients, and the red arrowhead nodes represent the bacteria that target the ingredients.

Retrieving from TCMID 2.0 (http://119.3.41.228:8000/tcmid), and then screening by DAVID Bioinformatics Resources 6.8 (NIAID/NIH; https://david.ncifcrf.gov/tools.jsp) analysis, we finally obtained 44 bacteria (i.e., *Enterococcus faecalis, Acinetobacter, Bacillus, Bacillus subtilis, Bifidobacterium, Citrobacter, Enterobacter, E. coli, Lactic acid bacteria, Lactobacillus acidophilus, Streptococcus, Shigella dysenteriae,* etc.). These bacteria were targeted by 6 bacterial genes from 6 ingredients of 6 herbs of CoTOL with STITCH combined-score more than 0.7 [20] (Figure 2). The entire ingredients and genes of CoTOL can be found in Supplementary Table S1.

### 4.2. Effect of CoTOL on Body Weight in Obese Hyperuricemic Mice

**Figure 3:**
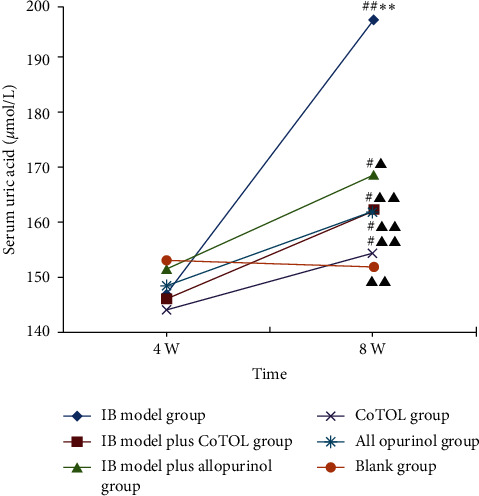
Body weight variations in each group of mice. After a one-week adaptation period, all mice were randomly divided into six groups: IB model group (obese hyperuricemia model inoculated with bacteria), IB model plus CoTOL group (IB model treated with CoTOL), IB model plus allopurinol group (IB model treated plus allopurinol), CoTOL group (obese hyperuricemia model treated with CoTOL), allopurinol group (obese hyperuricemia model treated with allopurinol), and blank group (C57BL/6J mice treated with water). Body weight was measured on the first day, 4^th^ and 8^th^ week. Compared with the first week of the same group, ^△△^*p* < 0.01. Compared with the 4^th^ week of the same group, ^#^*p* < 0.05, ^##^*p* < 0.01. Compared with the blank group, ^*∗*^*p* < 0.05.

To observe the effects of CoTOL on body weight, we constructed an obese mice model by feeding with high fat diet for 8 weeks. As shown in Figure 3, the weight of the IB model group mice increased significantly as compared to the blank group, which was more than 20%. Compared with the IB model group, the weights in IB model plus CoTOL group and CoTOL group showed a more significant decrease than those of IB model plus allopurinol group, and allopurinol group, respectively. The results showed that after CoTOL treatment for 4 weeks there was a definite effect on overweight which confirms its clinical use, possibly due to its regulating the intestinal flora.

### 4.3. Effect of CoTOL on Uric Acid in Obese Hyperuricemic Mice

**Figure 4:**
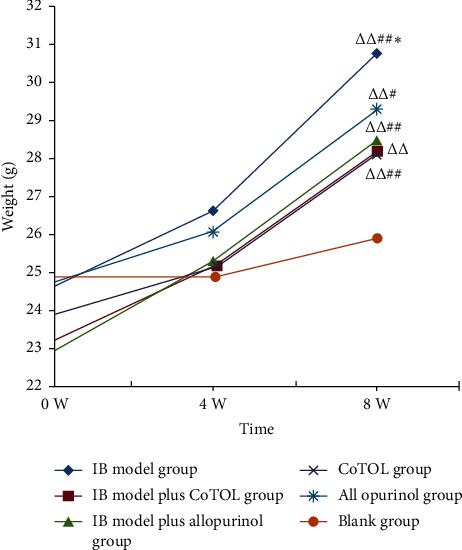
Serum uric acid levels in mice. Compared with the 4^th^ week of the same group, ^#^*p* < 0.05, ^##^*p* < 0.01. Compared with the blank group, ^*∗*^*p* < 0.05, ^*∗∗*^*p* < 0.01. Compared with IB model group, ^▲▲^*p* < 0.01.

It is well known that *Enterococcus faecalis (*i.e., *Streptococcus faecalis)* [7] and other microorganisms can produce xanthine oxidase and further decompose purine metabolism to produce uric acid. To observe the effect of *Streptococcus faecalis* and/or CoTOL on intestinal flora and uric acid, we established an obese hyperuricemic mice model by inoculating intragastrically with *Streptococcus faecalis* (a type of XOD-producing bacteria) and administration with PO (250 mg/kg). As shown in Figure 4, serum uric acid level in IB model group increased significantly (*p* < 0.01) at the 8^th^ week compared to blank group indicating that that XOD-producing bacteria (*streptococcus faecalis*) inoculation and PO injection caused a significant elevation in serum uric acid levels, whereas treatments with CoTOL or allopurinol decreased the uric acid levels compared to IB model group. The serum uric acid levels in IB model plus CoTOL group and CoTOL group were lower than those of IB model plus allopurinol group and allopurinol group. The results showed that CoTOL showed more anti-hyperuricemic activity higher than allopurinol, which may be attributed to its inhibitory effects on XOD-producing bacteria. Therefore, we further observed its effects on intestinal flora in obese hyperuricemic mice.

### 4.4. Effect of CoTOL on Intestinal Flora in Obese Hyperuricemic Mice

**Figure 5:**
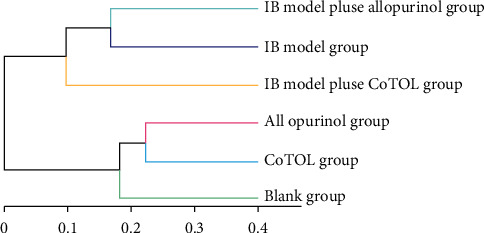
Clustering tree diagram of intestinal flora OTU in each group mice. The length of branch represents the distance between samples, and the more similar the sample will be, the closer it will be.

As shown in Figure 5, flora OTU of mice inoculated with or without inoculation of *Streptococcus faecalis* were divided into two categories. CoTOL group, allopurinol group, and blank group were clustered together without inoculation, whereas IB model group and IB model plus CoTOL or allopurinol group were clustered together; this implied that *Streptococcus faecalis* (XOD-producing bacteria) could significantly affect the intestinal flora of mice. In particular, the IB model plus CoTOL group was clustered alone and different from the other two groups (Figure 5). The results indicated that the intestinal flora of mice with or without CoTOL treatment was different. CoTOL showed a significant effect on intestinal flora in IB model mice.

### 4.5. Comparison of Intestinal Flora Structure and Abundance

**Figure 6:**
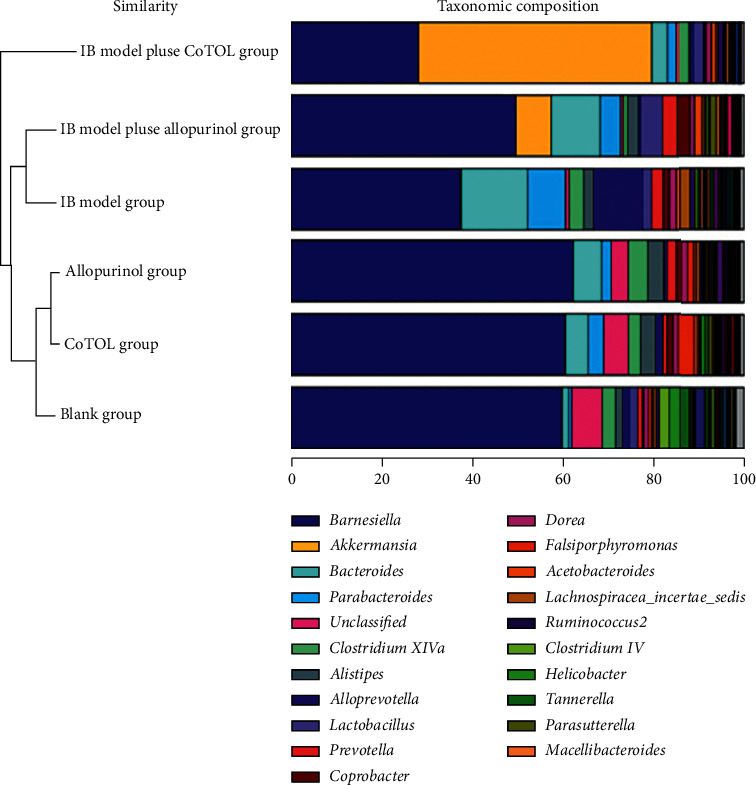
Structure and abundance of intestinal flora in the six groups. The colors correspond to the names of different intestinal flora species, and the width of the color bar represents the proportion of relative abundance of the species.

To further observe the effect of CoTOL on intestinal flora in obese hyperuricemic mice, we compared the structure and abundance of flora. As shown in Figure 6, the community structure and abundance of all bacteria in CoTOL group, allopurinol group, and blank group were very similar, mainly composed of *Barnesiella*, whereas the structure of IB model plus allopurinol group was similar to IB model group. The abundance of *Barnesiella* decreased in IB model group and that of *Bacteroides* and *Alloprevotella* increased. The results showed that XOD-producing bacteria (*Streptococcus faecalis*) could lead to the decrease of *Barnesiella* abundance and increase of *Bacteroides* and *Alloprevotella*. The abundance of *Akkermansia* in IB model plus CoTOL group increased significantly and its abundance was even higher than *Barnesiella* (Figure 6). Its flora structure obviously changed which is mainly composed of *Barnesiella and Akkermansia* due to CoTOL treatment. Thus, CoTOL treatment showed a significant effect on reducing weight and uric acid which may be attributed to its regulating the flora structure and abundance.

### 4.6. Analysis of the Correlation between Uric Acid and Flora

**Figure 7:**
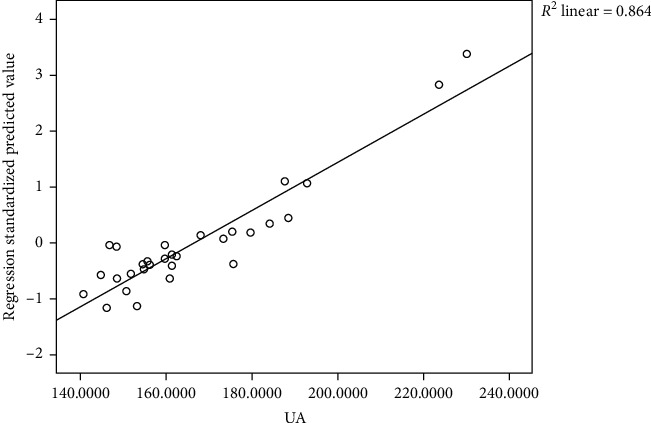
Correlation with flora and uric acid by linear regression line analysis. The strains of intestinal flora were positively correlated with uric acid (*R*-square = 0.864, *F* = 3.358, *p* = 0.027).

Based on the statistics of the OTU number, we selected different strains of intestinal flora with high abundance in the obese hyperuricemic model mice to analyze the correlation between intestinal flora and blood uric acid. By linear regression line analysis, we found that the strains of intestinal flora were correlated with uric acid (*R*-square = 0.864, *F* = 3.358, *p* = 0.027). And *Alloprevotella* (*r* = 0.600, *p* ≤ 0.01), *Bacteroides* (*r* = 0.631, *p* ≤ 0.01), *Lachnospiracea incertae sedis* (*r* = 0.739, *p* ≤ 0.01), and *Paracetoides* (*r* = 0.594, *p* ≤ 0.01) were significantly positively correlated with uric acid value of the obese hyperuricemic model mice, while *Barnesiella* (*r* = −0.443, *p* = 0.014) and *Tannerella* (*r* = −0.403, *p* = 0.027) were negatively correlated with uric acid value (Figure 7, Table 2).

### 4.7. Prediction and Analysis of Flora Function

**Figure 8:**
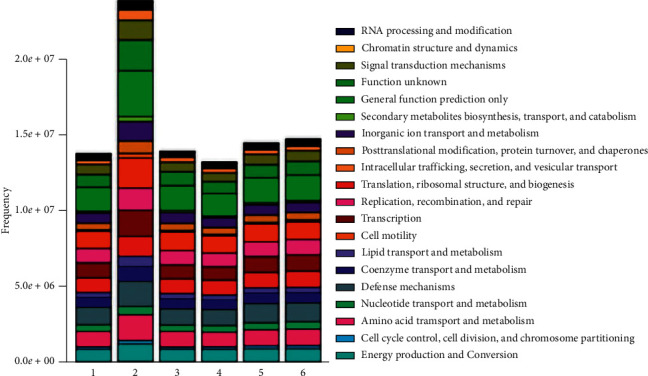
Histogram of flora functional distribution in each group. The numbers 1, 2, 3, 4, 5, and 6 refer to IB model group, IB model plus CoTOL group, IB model plus allopurinol group, CoTOL group, allopurinol group, and blank group, respectively. The colors correspond to the different flora function, and the width of the color bar represents the proportion of relative abundance of the species.

To further investigate the effect of CoTOL on flora, we analyzed and predicted the flora function among the different groups. Based on the macro-genomic sequencing data of flora function, we found that the flora function for IB model plus CoTOL group was significantly different from those of other groups (Figure 8). The difference mainly manifested in improving transport and metabolism of lipid, amino acid, and carbohydrate or energy production and conversion, etc.

## 5. Discussion

### 5.1. Effect on Overweight and Its Related Intestinal Flora

Hyperuricemia is caused by the disorder of purine metabolism in the human body. Its high incidence is mainly distributed in middle-aged and elderly men and postmenopausal women. It is closely related to many other metabolic syndromes, such as diabetes, hypertension, obesity, and other chronic diseases [21–23]. Obesity is also an important risk factor for hyperuricemia, gout, and insulin resistance, which is harmful to the development of hypertension, coronary heart disease, and diabetes mellitus [24]. The disorder of lipid metabolism is an important reason for elevation of uric acid levels caused by obesity [25, 26]. *Akkermansia muciniphila*, a kind of intestinal anaerobes, mainly grows in the intestinal mucinous layer of mammals [27].

Many studies confirmed that the *Akkermansia* is the key bacteria to reduce fat and its abundance is negatively correlated with obesity, diabetes, and other diseases [27, 28]. *Akkermansia muciniphila* is a promising probiotic improving the host metabolic functions and immune responses [29]. It could be regarded as a next-generation beneficial microbe via affecting glucose metabolism, lipid metabolism, and intestinal immunity [30]. We found that the flora structure obviously changed in IB model plus CoTOL group and the abundance of *Akkermansia* increased significantly even higher than that of *Barnesiella* due to CoTOL treatment (Figure 6). The flora structure in IB model plus CoTOL group was similar to IB model group. Thus, CoTOL exhibited a unique effect on reducing fat which may be attributed to its higher abundance of *Akkermansia.*

In the present study, we found that the body weight of the mice in the IB model group was the highest of all groups (Figure 3), which shows that inoculation with XOD-producing bacteria significantly caused a weight growth. CoTOL treatment group is lower than the IB model group and allopurinol group which showed that CoTOL treatment could inhibit the overweight. Moreover, we found that CoTOL treatment could change the community structure and abundance of intestinal flora. Flora function of IB model plus CoTOL group was different from those of other groups. The differences mainly manifested in the substance metabolism such as lipids, carbohydrates, and proteins according to the prediction of flora function (Figure 8). The enhancement of these metabolic functions plays an important role in improving obesity and maintaining the health. The results showed that CoTOL exhibited beneficial effect on reducing weight, which may be attributed to its regulating the intestinal flora. It is well known that overweight and obesity had strong associations with hyperuricemia. However, the underlying obesity metabolism, how CoTOL regulates intestinal flora, which species of flora, and effective ingredients works need to be further studied in our future research.

### 5.2. Relationship between Intestinal Flora and Hyperuricemia

Intestinal microbial flora is a complex ecosystem considered as the second genome of human body, which has an important role and influence on human health. Intestinal flora is closely related to the metabolism, nutrition, and immunity of the host. More and more studies have shown that intestinal flora disorders increase the risk of various chronic metabolic diseases, including obesity, diabetes, gout, hyperuricemia, and so on. Two-thirds of uric acid formed in the body is excreted through the kidney, and one third is excreted through the intestinal tract [31]. The decrease of uric acid decomposition function of intestinal microorganisms is an important cause of hyperuricemia and gout [11], while the effect of the decrease of intestinal excretion uric acid function on blood uric acid level has not been paid more attention. In recent years, many studies have found that the intestinal flora of patients with hyperuricemia is different from that of normal people. Compared with the intestinal flora of healthy people, hyperuricemia patients have a flora disorder, mainly manifested in the decrease of the number of *Lactobacillus*, while the number of *Bacillus*, *Escherichia coli*, and total aerobic bacteria increased [32, 33]. Xanthine oxidase plays an important role in the formation of uric acid. It was found that bacteria, *Streptomyces* and *Pseudomonas aeruginosa*, could produce xanthine oxidase and further decompose purine metabolism to uric acid. Therefore, XOD-producing bacteria may play an important role in the catabolism of serum uric acid.

Based on the correlation analysis, we found that *Alloprevotella, Bacteroides,Lachnosporiaincertae pedis*, and *Parabacteroides* had significant positive correlation with the uric acid levels of the obese hyperuricemic mice (Table 2). The two strains of *Barnesiella* and *Tannerella* were negatively correlated with the uric acid levels and they had a certain inhibitory effect on the uric acid in the obese hyperuricemic mice. The result of linear regression analysis indicated that the intestinal flora could have an important effect on the uric acid levels of the obese hyperuricemic mice.

*Barnesiella*, an anaerobic Gram-negative bacteria, can produce organic acid such as acetic acid [34], which significantly reduced in obese hyperuricemic mice. It is regarded as the most common dominant factor and plays an important role in maintaining the ecological balance of the intestinal flora.

*Bacteroide* is nonspore and specific anaerobe, which could be found in human and animal intestines, oral cavity, and upper respiratory tract. It has either probiotic or pathogenic effect. The number of *Bacteroide* in the feces of hyperuricemic patients increased significantly. *Alloprevotella* (*prevotella*) which belongs to Gram-negative anaerobes can trigger joint inflammation on the premise that autoimmunity exists*. Lachnospiraceae incertae sedis*, belonging to the family of *Trichomoniaceae*, is a butyric acid bacterium. Compared with lean mice, obese mice had more short-chain fatty acid, acetic acid, and butyric acid in cecum and intestinal bacteria involved in energy metabolism [35]. The elevation of butyric acid-producing bacteria in the intestinal tract of obese mice could lead to absorbing energy from the diet and developing into obesity. *Parabacteroides*, belonging to genus *Porphyromonas*, has a significant association with the bile acid, which plays an important role in the metabolism of fat [36]. In our study, we found that the bacteria had a significant effect on the elevation of serum uric acid in mice which may be related to bile acids.

### 5.3. Effect on Uric Acid and Intestinal Flora of the Mice

Uric acid-lowering drugs, such as allopurinol, may cause allergic reactions, bone marrow suppression, disorders in gastrointestinal tract and nervous system, and so on. TCMs are widely used to treat hyperuricemia, gout, and obesity with fewer side effects. Our previous studies [14, 37] showed that CoTOL could reduce serum uric acid levels in patients with chronic gout and prevent the recurrence of acute arthritis with no serious adverse effects. However, its underlying mechanism is not clear. In the present study, we used the network pharmacological analysis and found that 6 ingredients of 6 kinds of herbs in CoTOL could regulate 44 kinds of bacteria. The disorder of intestinal flora in hyperuricemic patients mainly manifested decreased dominant flora and increased pathogenic flora. Both *Streptomyces and Pseudomonas aeruginosa* can produce xanthine oxidase and further decompose purine metabolism into uric acid. According to our network pharmacological analysis, CoTOL had an effect on *Streptomyces and Pseudomonas aeruginosa*; thus it could affect the production of xanthine oxidase and reduce the production of uric acid. In addition, it has been found that many TCMs can exert their efficacy by inhibiting the activity of the key enzyme of uric acid synthesis (xanthine oxidase). For example, *Poria cocos* and *Alisma orientalis* had good effects on the decrease of serum uric acid in hyperuricemic mice by inhibiting the liver xanthine oxidase or by promoting the excretion of uric acid, respectively.

In the present study, we found that the uric acid levels in mice inoculated with *Streptococcus faecalis* were higher than those without inoculation. After treatment with CoTOL or allopurinol, the uric acid levels were significantly lower than those of IB model group. Based on the data of the flora abundance and structure, the abundance of *Barnesiella* decreased in IB model group and that of *Bacteroides* and *Alloprevotella* increased which showed that the XOD-producing bacteria (*Streptococcus faecalis*) could lead to the disorder of intestinal flora in obese hyperuricemic mice. The IB model plus CoTOL group was similar to the blank group with higher abundance of *Barnesiella* and lower those of *Bacteroides* and *Alloprevotella*. CoTOL had the effects on reducing the uric acid levels which may be correlated with its regulating intestinal flora.

## Figures and Tables

**Table 1:** CoTOL formula.

Herb	Part used	Dosage used (g)
*Glabrous Greenbrier Rhizome*	Rhizoma	30
*Dioscorea septemloba Thunb*	Rhizoma	30
*Curcuma Longa*	Rhizoma	12
*Parasitic loranthus*	Rhizoma	15
*Herba Siegesbeckiae*	Aboveground components	18
*Maydis stigma*	Interlobule	15
*Semen Coicis*	Seed	30
*Corydalis Rhizoma*	Tuber	18

**Table 2:** Analysis of the effect of intestinal flora on uric acid.

Variable	*R*	*p* value
UA	1	—
*Alloprevotella*	0.600	*p* ≤ 0.01 0.000
*Bacteroides*	0.631	*p* ≤ 0.01 0.001
*Lachnospiracea incertae sedis*	0.739	*p* ≤ 0.01 0.014
*Parabacteroides*	0.594	*p* ≤ 0.01 0.027
*Barnesiella*	−0.443	0.014
*Tannerella*	−0.403	0.027

## Data Availability

Data used to support the findings of this study are available from the corresponding author upon request.

## Abbreviations

CoTOL: Compound tufuling oral-liquid
UA: Uric acid
XOD/XDH: Xanthine oxidase/xanthine dehydrogenase
TCMID: Traditional Chinese medicines integrated database
DAVID: Database for annotation, visualization, and integrated discovery
OUT: Operational taxonomic units
TCM: Traditional Chinese medicine
ATP: Adenosine triphosphate
CFU: Colony-forming units
IB model group: Inoculated bacteria model group
IB model plus CoTOL group: IB model group intragastrically administrated with CoTOL
IB model plus allopurinol group: IB model group intragastrically administrated with allopurinol.

## Supplementary Materials

Supplementary Materials Supplementary Table S1: the entire ingredients and genes list of CoTOL.
