# Supplementary material for: Effect of a Traditional Chinese Medicine Formula (CoTOL) on Serum Uric Acid and Intestinal Flora in Obese Hyperuricemic Mice Inoculated with Intestinal Bacteria
Source: Evid Based Complement Alternat Med. 2020 Dec 23;2020:8831937. doi: 10.1155/2020/8831937 (PMC7775141; doi:10.1155/2020/8831937)
Supplement: Supplementary Materials — Supplementary Table S1: the entire ingredients and genes list of CoTOL. [file 8831937.f1.docx]

**Supplementary Table S1**

**The entire ingredients and genes list of CoTOL**

| Herbs | Ingredient Name | TARGETS | SCORE |
| --- | --- | --- | --- |
| *Semen Coicis* | arginine | Arg1 | 0.999 |
| *Semen Coicis* | copper | Atox1 | 0.999 |
| *Semen Coicis* | copper | Atp7a | 0.999 |
| *Semen Coicis* | copper | Atp7b | 0.999 |
| *Semen Coicis* | copper | Cp | 0.999 |
| *Semen Coicis* | calcium | F2 | 0.999 |
| *Semen Coicis* | arginine | Nos1 | 0.999 |
| *Semen Coicis* | arginine | Nos2 | 0.999 |
| *Semen Coicis* | arginine | Nos3 | 0.999 |
| *Semen Coicis* | calcium | PLA2G4A | 0.999 |
| *Semen Coicis* | calcium | PPP3CA | 0.999 |
| *Semen Coicis* | copper | Slc31a1 | 0.999 |
| *Semen Coicis* | zinc | Sod1 | 0.999 |
| *Semen Coicis* | calcium | F9 | 0.998 |
| *Semen Coicis* | stearic acid | fas | 0.998 |
| *Semen Coicis* | arginine | Gatm | 0.998 |
| *Semen Coicis* | calcium | GUCA1A | 0.998 |
| *Semen Coicis* | copper | Heph | 0.998 |
| *Semen Coicis* | calcium | MASP1 | 0.998 |
| *Semen Coicis* | calcium | MASP2 | 0.998 |
| *Semen Coicis* | arginine | Nags | 0.998 |
| *Semen Coicis* | calcium | NOS3 | 0.998 |
| *Semen Coicis* | calcium | PRKCA | 0.998 |
| *Semen Coicis* | calcium | PRKCG | 0.998 |
| *Semen Coicis* | arginine | Asl | 0.997 |
| *Semen Coicis* | palmitic acid | fasn | 0.997 |
| *Semen Coicis* | leucine | LARS | 0.997 |
| *Semen Coicis* | leucine | LARS2 | 0.996 |
| *Semen Coicis* | arginine | Ass1 | 0.995 |
| *Semen Coicis* | arginine | Slc7a1 | 0.995 |
| *Semen Coicis* | copper | Sod1 | 0.995 |
| *Semen Coicis* | copper | Sod3 | 0.995 |
| *Semen Coicis* | zinc | Uba52 | 0.995 |
| *Semen Coicis* | leucine | BCAT1 | 0.991 |
| *Semen Coicis* | leucine | BCAT2 | 0.991 |
| *Semen Coicis* | palmitic acid | acsl1 | 0.99 |
| *Semen Coicis* | zinc | Hsp90aa1 | 0.989 |
| *Semen Coicis* | myristic acid | Abl1 | 0.987 |
| *Semen Coicis* | zinc | Slc30a1 | 0.987 |
| *Semen Coicis* | zinc | Mtr | 0.986 |
| *Semen Coicis* | zinc | Nos3 | 0.984 |
| *Semen Coicis* | zinc | Slc30a2 | 0.984 |
| *Semen Coicis* | palmitic acid | acsbg2 | 0.983 |
| *Semen Coicis* | palmitic acid | acsl6 | 0.983 |
| *Semen Coicis* | zinc | Alad | 0.983 |
| *Semen Coicis* | zinc | Ngf | 0.983 |
| *Semen Coicis* | zinc | Cda | 0.982 |
| *Semen Coicis* | palmitic acid | fabp1 | 0.981 |
| *Semen Coicis* | palmitic acid | nos3 | 0.981 |
| *Semen Coicis* | palmitic acid | ppt1 | 0.981 |
| *Semen Coicis* | copper | Tf | 0.980 |
| *Semen Coicis* | leucine | EPRS | 0.978 |
| *Semen Coicis* | lysine | thrA | 0.970 |
| *Semen Coicis* | myristic acid | Tlr4 | 0.961 |
| *Semen Coicis* | myristic acid | Hnf4a | 0.960 |
| *Semen Coicis* | myristic acid | Gm2a | 0.957 |
| *Semen Coicis* | myristic acid | Ly96 | 0.957 |
| *Semen Coicis* | leucine | SLC6A15 | 0.953 |
| *Semen Coicis* | leucine | SLC6A18 | 0.948 |
| *Semen Coicis* | leucine | SLC6A19 | 0.948 |
| *Semen Coicis* | leucine | SLC6A14 | 0.947 |
| *Semen Coicis* | myristic acid | Abl2 | 0.939 |
| *Semen Coicis* | myristic acid | Rcvrn | 0.939 |
| *Semen Coicis* | leucine | OPLAH | 0.934 |
| *Semen Coicis* | myristic acid | Gcg | 0.911 |
| *Semen Coicis* | myristic acid | Hnf4g | 0.910 |
| *Semen Coicis* | phosphorus | SP7 | 0.800 |
| *Semen Coicis* | triolein | PNPLA3 | 0.762 |
| *Semen Coicis* | triolein | PNPLA4 | 0.755 |
| *Semen Coicis* | triolein | PNPLA2 | 0.753 |
| *Semen Coicis* | indene | UGT1A3 | 0.700 |
| *Semen Coicis* | indene | UGT2B7 | 0.700 |
| *Rhizoma Corydalis* | protoporphyrin | FECH | 0.999 |
| *Rhizoma Corydalis* | protoporphyrin | ALAS2 | 0.978 |
| *Rhizoma Corydalis* | canaline | OAT | 0.968 |
| *Rhizoma Corydalis* | berberine | Akt1 | 0.965 |
| *Rhizoma Corydalis* | protoporphyrin | CPOX | 0.959 |
| *Rhizoma Corydalis* | protoporphyrin | PPOX | 0.959 |
| *Rhizoma Corydalis* | glaucine | ABCB1 | 0.938 |
| *Rhizoma Corydalis* | berberine | Mapk1 | 0.934 |
| *Rhizoma Corydalis* | canaline | OTC | 0.914 |
| *Rhizoma Corydalis* | protoporphyrin | COX10 | 0.908 |
| *Rhizoma Corydalis* | berberine | Mapk3 | 0.896 |
| *Rhizoma Corydalis* | bicuculline | Slc12a5 | 0.891 |
| *Rhizoma Corydalis* | bicuculline | Gabrr3 | 0.882 |
| *Rhizoma Corydalis* | bicuculline | Gabrr1 | 0.878 |
| *Rhizoma Corydalis* | bicuculline | Gabbr2 | 0.873 |
| *Rhizoma Corydalis* | tetrahydropalmatine | DRD3 | 0.867 |
| *Rhizoma Corydalis* | bicuculline | Gabrr2 | 0.867 |
| *Rhizoma Corydalis* | berberine | Insr | 0.852 |
| *Rhizoma Corydalis* | bicuculline | Gabrb3 | 0.851 |
| *Rhizoma Corydalis* | berberine | Ldlr | 0.851 |
| *Rhizoma Corydalis* | bicuculline | Sst | 0.842 |
| *Rhizoma Corydalis* | berberine | Pcsk9 | 0.837 |
| *Rhizoma Corydalis* | bicuculline | Gabra1 | 0.836 |
| *Rhizoma Corydalis* | berberine | Slc2a4 | 0.834 |
| *Rhizoma Corydalis* | bicuculline | Gabrb2 | 0.833 |
| *Rhizoma Corydalis* | berberine | Stat3 | 0.833 |
| *Rhizoma Corydalis* | bicuculline | Gabrb1 | 0.832 |
| *Rhizoma Corydalis* | berberine | Ptgs2 | 0.832 |
| *Rhizoma Corydalis* | berberine | Ctsb | 0.827 |
| *Rhizoma Corydalis* | tetrahydropalmatine | DRD2 | 0.818 |
| *Rhizoma Corydalis* | protoporphyrin | FTL | 0.816 |
| *Rhizoma Corydalis* | stylopine | MPO | 0.816 |
| *Rhizoma Corydalis* | protoporphyrin | ADIPOQ | 0.800 |
| *Rhizoma Corydalis* | protopine | HRH1 | 0.800 |
| *Rhizoma Corydalis* | protopine | KIAA0101 | 0.800 |
| *Rhizoma Corydalis* | glaucine | MMP9 | 0.800 |
| *Rhizoma Corydalis* | coptisine | TNFSF11 | 0.800 |
| *Rhizoma Corydalis* | protopine | F2 | 0.786 |
| *Rhizoma Corydalis* | protoporphyrin | CASP8 | 0.700 |
| *Rhizoma Corydalis* | protoporphyrin | HIF1A | 0.700 |
| *Rhizoma Corydalis* | protoporphyrin | HSP90AA1 | 0.700 |
| *Curcuma longa* | ethanol | Adh1c | 0.999 |
| *Curcuma longa* | ethanol | Aldh1a1 | 0.999 |
| *Curcuma longa* | ethanol | Cyp2e1 | 0.999 |
| *Curcuma longa* | ethanol | Adh7 | 0.998 |
| *Curcuma longa* | formic acid | Lpl | 0.998 |
| *Curcuma longa* | formic acid | Pnlip | 0.998 |
| *Curcuma longa* | ethanol | Adh4 | 0.997 |
| *Curcuma longa* | lauric acid | fas | 0.997 |
| *Curcuma longa* | phenylacetaldehyde | AOC2 | 0.996 |
| *Curcuma longa* | phenylacetaldehyde | AOC3 | 0.996 |
| *Curcuma longa* | ethanol | Cat | 0.996 |
| *Curcuma longa* | formic acid | Cyp19a1 | 0.996 |
| *Curcuma longa* | formic acid | Gart | 0.996 |
| *Curcuma longa* | ethanol | Adh6 | 0.995 |
| *Curcuma longa* | formic acid | Asah2 | 0.994 |
| *Curcuma longa* | formic acid | Lipe | 0.994 |
| *Curcuma longa* | formic acid | Pdf | 0.993 |
| *Curcuma longa* | formic acid | Pla2g2a | 0.993 |
| *Curcuma longa* | formic acid | Pts | 0.993 |
| *Curcuma longa* | ethanol | Il6 | 0.990 |
| *Curcuma longa* | phenylacetaldehyde | MAOA | 0.989 |
| *Curcuma longa* | phenylacetaldehyde | MAOB | 0.989 |
| *Curcuma longa* | ethanol | Pomc | 0.988 |
| *Curcuma longa* | naphthalene | CYP2F1 | 0.986 |
| *Curcuma longa* | caprylic acid | lipB | 0.982 |
| *Curcuma longa* | octanoic acid | lipB | 0.982 |
| *Curcuma longa* | phenylacetaldehyde | ALDH3A1 | 0.980 |
| *Curcuma longa* | phenylacetaldehyde | ALDH3B1 | 0.971 |
| *Curcuma longa* | phenylacetaldehyde | ALDH1A3 | 0.962 |
| *Curcuma longa* | naphthalene | CYP1A2 | 0.962 |
| *Curcuma longa* | naphthalene | CYP1A1 | 0.951 |
| *Curcuma longa* | pentadecanoic acid | KALRN | 0.951 |
| *Curcuma longa* | naphthalene | NPY1R | 0.945 |
| *Curcuma longa* | naphthalene | CYP1B1 | 0.944 |
| *Curcuma longa* | naphthalene | CYP2C18 | 0.942 |
| *Curcuma longa* | naphthalene | CYP2C8 | 0.942 |
| *Curcuma longa* | naphthalene | CYP2D6 | 0.942 |
| *Curcuma longa* | naphthalene | CYP2J2 | 0.942 |
| *Curcuma longa* | phenylacetaldehyde | ADH6 | 0.923 |
| *Curcuma longa* | phenylacetaldehyde | ADH1A | 0.922 |
| *Curcuma longa* | phenylacetaldehyde | ADH4 | 0.922 |
| *Curcuma longa* | pentadecanoic acid | F2RL1 | 0.921 |
| *Curcuma longa* | naphthalene | CYP2A13 | 0.920 |
| *Curcuma longa* | pentadecanoic acid | OXT | 0.902 |
| *Curcuma longa* | pentadecanoic acid | GNRHR | 0.894 |
| *Curcuma longa* | pentadecanoic acid | HRH1 | 0.894 |
| *Curcuma longa* | pentadecanoic acid | OXTR | 0.894 |
| *Curcuma longa* | pentadecanoic acid | QRFPR | 0.894 |
| *Curcuma longa* | pentadecanoic acid | TACR1 | 0.894 |
| *Curcuma longa* | n-undecane | bop | 0.845 |
| *Curcuma longa* | tetramethylpyrazine | Tnf | 0.828 |
| *Curcuma longa* | heptanol | Panx1 | 0.815 |
| *Curcuma longa* | p-cymene | FTL | 0.800 |
| *Curcuma longa* | tetramethylpyrazine | Epas1 | 0.733 |
| *Curcuma longa* | alpha-terpineol | CASP8 | 0.723 |
| *Curcuma longa* | capraldehyde | ALDH1A2 | 0.718 |
| *Curcuma longa* | tetramethylpyrazine | Cftr | 0.700 |
| *Curcuma longa* | germacron | CYP1A2 | 0.700 |
| *Curcuma longa* | germacron | CYP2B6 | 0.700 |
| *Curcuma longa* | germacron | CYP2C19 | 0.700 |
| *Curcuma longa* | germacron | CYP2C9 | 0.700 |
| *Curcuma longa* | germacron | CYP2D6 | 0.700 |
| *Curcuma longa* | germacron | CYP3A4 | 0.700 |
| *Curcuma longa* | germacron | NR1I2 | 0.700 |
| *poison yam* | palmitic acid | fasn | 0.997 |
| *poison yam* | emodin | CSNK2A1 | 0.992 |
| *poison yam* | palmitic acid | acsl1 | 0.990 |
| *poison yam* | 7-epitaxol | CYP2C8 | 0.987 |
| *poison yam* | palmitic acid | cpt2 | 0.984 |
| *poison yam* | palmitic acid | acsbg2 | 0.983 |
| *poison yam* | palmitic acid | acsl6 | 0.983 |
| *poison yam* | 7-epitaxol | EGFR | 0.982 |
| *poison yam* | palmitic acid | fabp1 | 0.981 |
| *poison yam* | palmitic acid | nos3 | 0.981 |
| *poison yam* | palmitic acid | ppt1 | 0.981 |
| *poison yam* | palmitic acid | acsl4 | 0.979 |
| *poison yam* | 7-epitaxol | TP53 | 0.978 |
| *poison yam* | 7-epitaxol | CYP3A4 | 0.977 |
| *poison yam* | 7-epitaxol | NR1I2 | 0.974 |
| *poison yam* | 7-epitaxol | CASP3 | 0.972 |
| *poison yam* | 7-epitaxol | CDK1 | 0.970 |
| *poison yam* | 7-epitaxol | CCNB1 | 0.968 |
| *poison yam* | 7-epitaxol | AKT1 | 0.965 |
| *poison yam* | 7-epitaxol | MAPK8 | 0.960 |
| *poison yam* | diosgenin | Mmp2 | 0.943 |
| *poison yam* | diosgenin | Mmp9 | 0.911 |
| *poison yam* | 5-HMF | ADH7 | 0.910 |
| *poison yam* | emodin | CYP1B1 | 0.824 |
| *poison yam* | emodin | VEGFA | 0.824 |
| *poison yam* | emodin | ERBB2 | 0.822 |
| *poison yam* | emodin | TP53 | 0.819 |
| *poison yam* | emodin | LCK | 0.818 |
| *poison yam* | emodin | TNF | 0.818 |
| *poison yam* | emodin | MCL1 | 0.811 |
| *poison yam* | 5-HMF | ADH6 | 0.803 |
| *poison yam* | emodin | CXCR4 | 0.800 |
| *poison yam* | diosgenin | Timp2 | 0.789 |
| *poison yam* | diosgenin | Vegfa | 0.789 |
| *poison yam* | diosgenin | Cflar | 0.786 |
| *poison yam* | diosgenin | Hgf | 0.786 |
| *poison yam* | diosgenin | Ptgs2 | 0.786 |
| *poison yam* | diosgenin | Shh | 0.786 |
| *poison yam* | diosgenin | Lipc | 0.741 |
| *poison yam* | diosgenin | Tnfrsf10b | 0.733 |
| *Loranthus parasiticus* | oleanolic acid | Nqo1 | 0.949 |
| *Loranthus parasiticus* | oleanolic acid | Ugt1a1 | 0.909 |
| *Loranthus parasiticus* | oleanolic acid | Ugt1a8 | 0.909 |
| *Loranthus parasiticus* | oleanolic acid | Ugt1a9 | 0.909 |
| *Loranthus parasiticus* | oleanolic acid | Nfe2l2 | 0.906 |
| *Loranthus parasiticus* | oleanolic acid | Ugt3a1 | 0.900 |
| *Loranthus parasiticus* | oleanolic acid | Ugt3a2 | 0.900 |
| *Loranthus parasiticus* | quercitrin | CYP3A4 | 0.846 |
| *Loranthus parasiticus* | oleanolic acid | Ppara | 0.826 |
| *Loranthus parasiticus* | quercitrin | CYP1A1 | 0.778 |
| *Loranthus parasiticus* | quercitrin | CYP1B1 | 0.761 |
| *Loranthus parasiticus* | quercitrin | CYP3A5 | 0.742 |
| *Loranthus parasiticus* | quercitrin | RPS6KA1 | 0.741 |
| *Herba Siegesbeckiae* | stearin | LPL | 0.952 |
| *Herba Siegesbeckiae* | na | SULT4A1 | 0.933 |
| *Herba Siegesbeckiae* | stearin | PLIN1 | 0.856 |
| *Herba Siegesbeckiae* | kirenol | ANXA1 | 0.828 |
| *Herba Siegesbeckiae* | stearin | DGAT2 | 0.815 |
| *Herba Siegesbeckiae* | stearin | COX5A | 0.794 |
| *Herba Siegesbeckiae* | stearin | COX6A2 | 0.794 |
| *Herba Siegesbeckiae* | stearin | COX6C | 0.794 |
| *Herba Siegesbeckiae* | stearin | COX7A1 | 0.794 |
| *Herba Siegesbeckiae* | na | NAT1 | 0.752 |
| *Herba Siegesbeckiae* | na | SULT1A1 | 0.735 |
| *Herba Siegesbeckiae* | na | NAT2 | 0.728 |
| *Herba Siegesbeckiae* | na | CYP1A2 | 0.722 |
| *Herba Siegesbeckiae* | na | SULT1A2 | 0.700 |
| *Herba Siegesbeckiae* | na | SULT1A3 | 0.700 |
| *Herba Siegesbeckiae* | na | SULT1C2 | 0.700 |
| *Glabrous Greenbrier Rhizome* | resveratrol | Sirt1 | 0.999 |
| *Glabrous Greenbrier Rhizome* | trans-resveratrol | Sirt1 | 0.999 |
| *Glabrous Greenbrier Rhizome* | linoleic acid | ACSL1 | 0.994 |
| *Glabrous Greenbrier Rhizome* | linoleic acid | FADS2 | 0.992 |
| *Glabrous Greenbrier Rhizome* | resveratrol | Akt1 | 0.991 |
| *Glabrous Greenbrier Rhizome* | trans-resveratrol | Akt1 | 0.991 |
| *Glabrous Greenbrier Rhizome* | resveratrol | Pparg | 0.989 |
| *Glabrous Greenbrier Rhizome* | trans-resveratrol | Pparg | 0.989 |
| *Glabrous Greenbrier Rhizome* | resveratrol | Sirt5 | 0.988 |
| *Glabrous Greenbrier Rhizome* | trans-resveratrol | Sirt5 | 0.988 |
| *Glabrous Greenbrier Rhizome* | resveratrol | Esr1 | 0.987 |
| *Glabrous Greenbrier Rhizome* | trans-resveratrol | Esr1 | 0.987 |
| *Glabrous Greenbrier Rhizome* | linoleic acid | ANGPTL4 | 0.977 |
| *Glabrous Greenbrier Rhizome* | linoleic acid | APOA1 | 0.977 |
| *Glabrous Greenbrier Rhizome* | linoleic acid | PPARGC1A | 0.977 |
| *Glabrous Greenbrier Rhizome* | linoleic acid | ACSL4 | 0.976 |
| *Glabrous Greenbrier Rhizome* | linoleic acid | PPARA | 0.973 |
| *Glabrous Greenbrier Rhizome* | resveratrol | Nampt | 0.971 |
| *Glabrous Greenbrier Rhizome* | trans-resveratrol | Nampt | 0.971 |
| *Glabrous Greenbrier Rhizome* | resveratrol | Hmox1 | 0.970 |
| *Glabrous Greenbrier Rhizome* | trans-resveratrol | Hmox1 | 0.970 |
| *Glabrous Greenbrier Rhizome* | resveratrol | Vegfa | 0.970 |
| *Glabrous Greenbrier Rhizome* | trans-resveratrol | Vegfa | 0.970 |
| *Glabrous Greenbrier Rhizome* | resveratrol | Ptgs2 | 0.969 |
| *Glabrous Greenbrier Rhizome* | trans-resveratrol | Ptgs2 | 0.969 |
| *Glabrous Greenbrier Rhizome* | dihydroresveratrol | LTA4H | 0.962 |
| *Glabrous Greenbrier Rhizome* | hexose | GCG | 0.886 |
| *Glabrous Greenbrier Rhizome* | hexose | HK1 | 0.836 |
| *Glabrous Greenbrier Rhizome* | hexose | HK2 | 0.836 |
| *Glabrous Greenbrier Rhizome* | hexose | HK3 | 0.831 |
| *Glabrous Greenbrier Rhizome* | hexose | CCND1 | 0.818 |
| *Glabrous Greenbrier Rhizome* | hexose | HKDC1 | 0.815 |
| *Glabrous Greenbrier Rhizome* | hexose | PRL | 0.800 |
| *Stigma Maydis* | inositol | Slc2a13 | 0.997 |
| *Stigma Maydis* | inositol | Impa1 | 0.993 |
| *Stigma Maydis* | inositol | Slc5a11 | 0.993 |
| *Stigma Maydis* | inositol | Cdipt | 0.991 |
| *Stigma Maydis* | inositol | Miox | 0.991 |
| *Stigma Maydis* | inositol | Impa2 | 0.990 |
| *Stigma Maydis* | inositol | Isyna1 | 0.989 |
| *Stigma Maydis* | inositol | Impad1 | 0.980 |
| *Stigma Maydis* | inositol | Cds1 | 0.978 |
| *Stigma Maydis* | vitamin k | NQO1 | 0.929 |
| *Stigma Maydis* | inositol | Inpp1 | 0.914 |
| *Stigma Maydis* | vitamin k | F2 | 0.811 |
